# The Bacillus of Typhoid Fever

**Published:** 1886-08

**Authors:** George W. Lewis


THE BUFFALO MEDICAL AND SURGICAL JOURNAL

Vol. XXVII. AUGUST, 1886. No. 1.

### Original Communications.

### *THE BACILLUS OF TYPHOID FEVER.*

Some Observations on its Life History — A Series of Experiments, with their Results.

By GEORGE W. LEWIS, Jr., A. M., M. D.

Having recently completed a series of experiments upon the bacillus of typhoid fever, I will briefly describe the methods employed and their results.

The patient was a young man, 17 years of age, who had passed through the various stages of the disease with unusual severity. The symptoms of each stage were well marked, and there was left no doubt as to the diagnosis. During the third week, the case was complicated by capillary bronchitis; the patient dying six days later. The post-mortem confirmed the diagnosis, and immediately cultivation experiments were begun.

The cultivating media employed were food-gelatine, blood serum and the cut surfaces of boiled potatoes. I will say here that I made use of these media, not because they are the only ones in which micro-organisms can be artificially cultivated, but because they afforded a solid substance, in contradistinction to the many nutrient liquids which have been employed so much, in years past, for this purpose. The advantage of the former is obvious, in that it enables the operator to obtain a pure culture in a comparatively short time, while the latter precludes the possibility of completely isolating any one particular organism. The assertion, which is so stoutly maintained by many anti-bacteriologists, that the myriad forms of bacteria are but stages in the development of one, or at the most, only a few species, seems to me to have grown out of the fact that they were unable to study the life-history of their cultivations, on account of the liquid character of the media. For example, suppose a drop of liquid, containing several kinds of bacteria, be introduced into a liquid medium; it is manifest that we have a mixed culture from the outset, and that all efforts to colonize the species would be in vain. It has been established, experimentally, tnat certain kinds of bacteria cannot develop in the presence of others, and that some require several days before their growth is perceptible, while others reach their maximum in a single day. Under these conditions, it is but natural that in the struggle for existence, the predominance of one form over another, especially the predominance of a pathogenic over a non-pathogenic, should result in the actual manifestation of disease. The tendency of all bacteria is to colony formation, and in liquid media this is rendered practically impossible. If, now, we can furnish a substance which will remain solid during the cultivating process, we not only act in accordance with a characteristic peculiar to all bacteria, but we are thereby enabled to study more minutely the various stages in their life-history, and, at the same time, render impossible the destruction of any of the existing forms of bacteria, because of their complete isolation.

Food-gelatine is best prepared by taking one pound of beef, as free from fat as possible, and chopping it into fine particles. Place it in a clean flask, and add one litre of distilled water. After shaking thoroughly, allow it to stand one night in an ice-chest. In the meantime, 100 test-tubes should be washed, rinsed with alcohol, and heated over a gas jet until thoroughly dry. Fit a wad of absorbent cotton into the mouth of each tube, and again sterilize over a Bunsen burner until the white cotton becomes a light-brown. Subject the tubes now to a temperature of 150° C. in a hot air sterilizer for one hour. During this interval, the meat infusion should be strained through a towel of coarse texture. As a result of the straining, there is usually about one litre. If it falls short of this, enough distilled water may be used to make up the amount. To this red juice add 10 grammes of peptonum siccum, 5 grammes of common salt, and 100 grammes of best stick gelatine. A wet towel wrapped about the beaker will tend to prevent its cracking during the gentle heating, which must needs be given it in order to thoroughly dissolve the gelatine. The re-action should be slightly alkaline, and is best taken upon litmus paper. A concentrated solution of soda may be added, drop by drop, until the red litmus paper becomes faintly blue. If originally too alkaline, it may be neutralized by the use of a little lactic acid. The mixture should now be heated for three-quarters of an hour in a water-bath, and then filtered through a thickness of filter-paper, made in the form of a tunnel. The food-gelatine is now ready for the sterilized test-tubes, which should be filled to about one-third of their length. Placed together in a dish, they must be sterilized for twenty minutes for five successive days, at a temperature of 100° C. Any cloudiness of the nourishing medium may be corrected by adding the white and shell of an egg, beaten up together. Of course, this necessitates the pouring back of the nutrient gelatine into the beaker, and boiling as before, being careful to repeat all the sterilizing precautions.

On June 2d, I inoculated a tube of the gelatine with blood from an inflamed Peyer's gland, another from the spleen, and still another from a mesenteric gland. Forty-eight hours later, I took three more tubes of food-gelatine, and liquified them by allowing them to stand for a few minutes in a pan of hot water I then inoculated, by means of a fine platinum needle, one of the tubes thus liquified with a single needleful of the growth obtained forty-eight hours after the original inoculation. After gently shaking the tube in order to thoroughly distribute the organisms, I inoculated a second tube from the first; this time, however, dipping the needle in three times instead of once. This was done on account of the already attenuated form of the first inoculation. A still higher attenuation was made by inoculating a third tube with five needlefuls from the second. Merely as a confirmatory measure, I followed out the same process on the blood taken from the spleen and mesenteric gland. Each tube was labeled with its attenuation and source, and replaced in the pan of warm water to maintain the liquid state.

I then sterilized, over a Bunsen burner, several pieces of ordinary window glass, 6x8 inches, and, in handling them afterwards, was careful to use thoroughly sterilized forceps to prevent my fingers from coming in contact with the plates. Two soup plates and two Bell jars were washed in sublimate solution, (1 to 1,000,) and after lining the soup plates with three or four thicknesses of filter paper, saturated with the same solution, I placed three of the sterilized glass plates, one upon another, with intervening bridges, and covered the whole with a Bell jar. The tubes, to be called for convenience 1, 2 and 3, which have been inoculated from the tube containing the original blood, were then poured upon the sterilized glass plates, No. 1 upon the first plate, beginning from the bottom, and so on, up. Care was taken to spread the gelatine evenly with a sterilized glass rod, so that it might become uniformly hard. The Bell jar was replaced and labeled with the details of the experiment, after which it was deposited in a shaded part of the room, to favor, and hasten somewhat, the colony formation.

At the end of twenty-four hours, colonization was detected only by using a magnifying power of 100 diameters. Plate No. 1, moreover, was the only one which manifested any trustworthy signs, even at the end of thirty-six hours. The predominating colony at this time was nothing more than a yellowbrownish speck, almost circular in form, and exhibiting in parts the so-called saw-edged appearance. A large proportion of bacteria liquify the gelatine as colonization progresses; this organism, however, does not, and this accounts in a degree, I think, for the somewhat longer time required for the satisfactory detection of the colonies. At the end of forty-eight hours, plates No. 2 and 3 showed a few colonies, and at sixty hours, although not very numerous, they were plainly visible to the unaided eye.

I then placed plate No. 1 upon the stage of the microscope, and with a fine platinum needle, slightly bent at the extremity into a miniature hook, and with the hand steadied by resting the little finger on the stage of the microscope, I fished a hookful out of the centre of one of the characteristic colonies. This procedure was accomplished under a magnifyii g power of 100 diameters. Immediately I inoculated a tube of fresh food-gelatine with my "catch," and would naturally expect as a result a pure cultivation of the organism. I also made several cover-glass preparations from a "catch" obtained in the same way from a similar colony, and colored them with a watery solution of fuchsine. A far more satisfactory, although somewhat longer, method of staining is with Bismarck brown, either a concentrated or a watery solution. This bacillus does not take an aniline staining at all easily.

I was now prepared to study the organism itself, and found several extremely interesting peculiarities, not only in its manner of growth, but in its individual appearance. The ends are blunt and rounded, and I detected a slight constriction in the middle where the organism measured 2-10 of a mm. in breadth. They commonly arrange themselves in filaments, sometimes extending across the entire field. Spore formation was present to a greater or less extent in all the preparations, and was always noticed at the ends of the rods. The threads were very active until overcome by the coloring material.

In the course of twenty-four hours, the inoculation streak in the test-tube of food-gelatine became visible, and, at the end of forty-eight hours, a distinct whitish growth had developed. Solid blood serum affords a most fertile soil on which to cultivate the bacillus of typhoid fever. It is prepared in the following manner: After thoroughly washing several glass jars with sublimate solution, (1 to 1,000,) and then with alcohol, they should finally be rinsed with ether. This evaporates and leaves the jars ready for use. I think on many accounts the sheep is the most accessible, and withal the best, animal from which to draw the blood. The part selected must be washed with sublimate, and the operation conducted with sterilized knives. It is better not to make use of the first show of blood. The vessels should be filled nearly full, and the glass stoppers replaced as soon as possible. In order to hasten the separation of the clot, the jars may be placed in an ice chest for thirty-six hours. The clear serum can then be poured into sterilized test-tubes, prepared in the same manner as for food-gelatine. Only about one-third of the length of the test-tube should be utilized. They must now be subjected to a heat of 58° C. for one hour in the Koch sterilizer. This is repeated for six days, the temperature on the last day being gradually raised to 60° C. In order to thoroughly solidify the serum, the tube must be allowed to rest at an angle of say 30°, thus spreading the serum over a greater surface. After being heated occasionally at 68° or 70° C., the serum is characterized by being hard, transparent, and of a color resembling straw.

To inoculate the blood-serum, the same instruments and precautions are needed as with food-gelatine. A fine platinum needle, thoroughly sterilized, is dipped into a pure culture, and then drawn in streaks over the solid serum. After thirty-six hours, a whitish and somewhat elevated layer makes its appearance. This continues to increase until almost the entire surface of the serum is covered.

The cut surface of a boiled potato is a suitable culture medium for almost all bacteria, and so was tried in the case of this organism. The potatoes should be sound and have few "eyes;" and after soaking for one half hour in sublimate solution, the eyes should be carefully removed with the point of a knife, and the whole potato scrubbed with a brush. They are then cooked for three-quarters of an hour in the steam-sterilizer, and should be allowed to remain there until needed for use. Several Bell jars are then washed in sublimate solution, and placed in as many soup-plates, lined with filter paper and saturated with the same solution. Knife-blades, for the purpose of cutting the potatoes in halves, must be well sterilized over a Bunsen burner, and every precaution taken not to touch the cut surface of the potato with the fingers. Once sterilized, the blades must be carefully watched, lest they come in contact with some unsterilized medium. After cutting the potatoes, they are to be placed upon the damp filter paper, under the Bell jar, with the cut surface up. At the end of thirty-six hours, there was a luxuriant growth of a whitish color. The temperature best suited to its development on potatoes is 98.6 F.

Of six mice and two rabbits inoculated with a pure culture of this organism, four of the mice and both of the rabbits died inside of nine days. The only pronounced symptom—and this was common to all except one rabbit—was the constant and exhausting diarrhœa. Upon examination, no marked changes were detected in the spleen or in any of the intestinal glands.

